# Common and rare variants associated with kidney stones and biochemical traits

**DOI:** 10.1038/ncomms8975

**Published:** 2015-08-14

**Authors:** Asmundur Oddsson, Patrick Sulem, Hannes Helgason, Vidar O. Edvardsson, Gudmar Thorleifsson, Gardar Sveinbjörnsson, Eik Haraldsdottir, Gudmundur I. Eyjolfsson, Olof Sigurdardottir, Isleifur Olafsson, Gisli Masson, Hilma Holm, Daniel F. Gudbjartsson, Unnur Thorsteinsdottir, Olafur S. Indridason, Runolfur Palsson, Kari Stefansson

**Affiliations:** 1 deCODE genetics/Amgen, Inc., Reykjavik 101, Iceland; 2 School of Engineering and Natural Sciences, University of Iceland, Reykjavik 101, Iceland; 3 Children's Medical Center, Landspitali—The National University Hospital of Iceland, Reykjavik 101, Iceland; 4 Faculty of Medicine, University of Iceland, Reykjavik 101, Iceland; 5 The Rare Kidney Stone Consortium, Mayo Clinic, Rochester, Minnesota, USA; 6 Icelandic Medical Center (Laeknasetrid), Laboratory in Mjodd (RAM), Reykjavik 109, Iceland; 7 Department of Clinical Biochemistry, Akureyri Hospital, Akureyri, 600, Iceland; 8 Department of Clinical Biochemistry, Landspitali University Hospital, Reykjavik 101, Iceland; 9 Division of Nephrology, Internal Medicine Services, Landspitali—The National University Hospital of Iceland, Reykjavik Iceland

## Abstract

Kidney stone disease is a complex disorder with a strong genetic component. We conducted a genome-wide association study of 28.3 million sequence variants detected through whole-genome sequencing of 2,636 Icelanders that were imputed into 5,419 kidney stone cases, including 2,172 cases with a history of recurrent kidney stones, and 279,870 controls. We identify sequence variants associating with kidney stones at *ALPL* (rs1256328[T], odds ratio (OR)=1.21, *P*=5.8 × 10^−10^) and a suggestive association at *CASR* (rs7627468[A], OR=1.16, *P*=2.0 × 10^−8^). Focusing our analysis on coding sequence variants in 63 genes with preferential kidney expression we identify two rare missense variants SLC34A1 p.Tyr489Cys (OR=2.38, *P*=2.8 × 10^−5^) and TRPV5 p.Leu530Arg (OR=3.62, *P*=4.1 × 10^−5^) associating with recurrent kidney stones. We also observe associations of the identified kidney stone variants with biochemical traits in a large population set, indicating potential biological mechanism.

## Abstract (web summary)

Kidney stone formation is influenced by genetic factors and recurrent stone formation places a significant burden on health care systems. Here Oddsson *et al.* perform a large-scale genome-wide association study and uncover new genetic variants associated with kidney stone susceptibility and associated biochemical traits.

## Graphical abstract

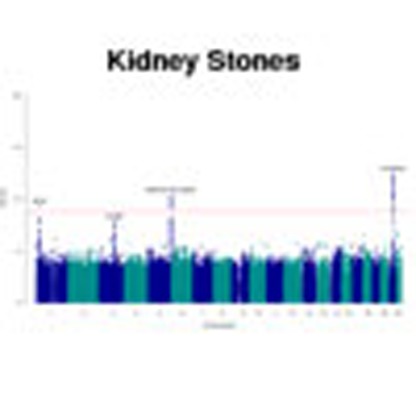

The lifetime risk of kidney stones is 8.8% in the United States^1^ with an estimated recurrence rate of 14% after 1 year and 35% after 5 years^2^, placing a significant burden on the health care system^1^. In Iceland, the prevalence in individuals older than 70 years is 10.1% for men and 4.2% for women^3^. Kidney stones form when urine becomes supersaturated with salts such as calcium oxalate or calcium phosphate and when urine concentrations of natural inhibitors of stone formation such as citrate, magnesium, pyrophosphate, uromodulin and osteopontin are low^4^,^5^. Calcium oxalate and calcium phosphate are the most common (∼80%) constituents of kidney stones^5^. The mechanism of stone formation involves both environmental factors such as diet^6^ and genetic traits. Individuals with a family history of kidney stones are at a greater risk than others of developing the condition. Recent studies estimate that up to 65% of kidney stone formers have a family history of kidney stones^7^,^8^,^9^ and both twin^10^ and genealogy^11^ studies have reported strong heritability of kidney stone disease. Candidate gene association studies have attempted to assess the role of several genes involved in calcium homeostasis on kidney stone formation^12^. These studies have been limited by sample size, the results are conflicting and they do not consider a broad spectrum of sequence variants.

We previously published a genome-wide association study (GWAS) of kidney stone disease, testing a total of 303,120 variants in 1,507 cases and 34,033 controls from Iceland^13^. Following replication in additional cases and controls from Iceland and the Netherlands, we reported a genome-wide significant association of the *CLDN14* locus, encoding the tight junction protein Claudin-14, on 21q22.13 with kidney stones (rs219780[C], allele frequency=79.20%, odds ratio (OR)=1.25, *P*=4.0 × 10^−12^). A GWAS in the Japanese population involving 904 cases and 7,471 controls reported three loci associated with kidney stones following replication in additional cases and controls, *RGS14*-*SLC34A1*, encoding regulator of G-protein signalling 14 and the Na/Pi co-transporter solute carrier family 34 member 1, on 5q35.3 (rs11746443[A], OR=1.19, *P*=8.5 × 10^−2^), *INMT*-*FAM188B*-*AQP1*, a locus including the gene encoding aquaporin 1, on 7p14.3 (rs1000597[C], OR=1.22, *P*=2.2 × 10^−14^), and *DGKH*, encoding diacylglycerol kinase eta, on 13q14.1 (rs4142110[C], OR=1.14, *P*=4.6 × 10^−9^)^14^.

In the current study, we have substantially increased the sample size from our previous study^13^ (*N* cases=5,419; *N* controls=279,870). We were also able to increase the number of sequence variants tested by performing GWAS on imputed genotypes of sequence variants identified through whole-genome sequencing of 2,636 Icelanders, yielding a more extensive coverage of the genome. In addition to discovering associations with kidney stones, we assessed the association of these variants to 13 biochemical traits involved in calcium–phosphate metabolism, purine metabolism, kidney function, acid–base and ion homeostasis in a large population set.

## Results

### Main findings

In our previous work, we sequenced the whole genomes of 2,636 Icelanders^15^,^16^ (median sequencing depth of 23 × ) yielding 28.3 million sequence variants. Subsequently, we imputed these variants assisted by long-range haplotype phasing into 98,721 Icelanders genotyped with Illumina SNP chips^17^,^18^. Using Icelandic genealogy data, we also calculated genotype probabilities of untyped close relatives of chip-typed individuals^19^. We examined the association between sequence variants and kidney stones in 5,419 Icelandic kidney stone formers^3^ (2,979 chip-typed and 2,440 chip-typed first- or second-degree relatives) including 2,172 recurrent kidney stone formers (see Methods for definition) and 279,870 controls (88,266 chip-typed and 191,604 chip-typed first- or second-degree relatives). We assessed the association of kidney stone associated sequence variants with biochemical parameters involved in calcium–phosphate metabolism (serum calcium, *N*=114,489; serum ionized calcium, *N*=18,516; serum phosphate, *N*=51,056; parathyroid hormone (PTH), *N*=15,541; 25-hydroxy vitamin D, *N*=7,544; alkaline phosphatase (ALP), *N*=126,060), purine metabolism (serum uric acid, *N*=56,025), acid–base homeostasis (serum bicarbonate (*N*=44,511), kidney function (serum creatinine, *N*=195,933) and ion homeostasis (serum chloride, *N*=92,938; serum magnesium, *N*=37,188; serum potassium, *N*=201,720; serum sodium, *N*=198,119).

**Figure 1:**
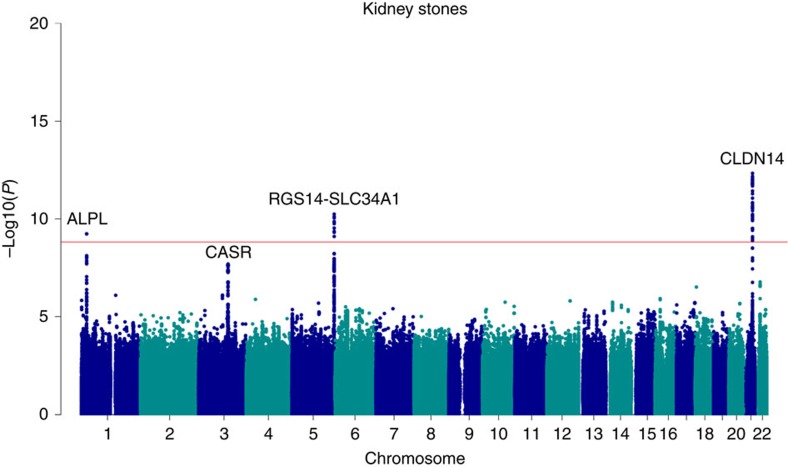
Manhattan plot showing individual *P* values against genomic positions for associations with kidney stones Genome-wide association analysis of kidney stones yielded four loci (*CLDN14*, *RGS14-SLC34A1*, *ALPL* and *CASR*) that influence kidney stones in Iceland (cases: 5,419, controlls: 279,870 ). Logistic regression was used for association testing between sequence variants and disease, treating disease status as the response and genotype counts as covariates. For the plot, the –log10 *P* values (*y* axis) of sequence variants are shown according to thier chromosomal position (*x* axis). The red line indicates the threshold for genome-wide statistical significance, which takes into account the effects of multiple testing (*P*=0.05/28.3 million=1.8 × 10^−9^).

In this study, we found variants at three loci associated with kidney stones at a genome-wide significance level (0.05/28.3 million=1.8 × 10^−9^) and one additional locus with suggestive association (Fig. 1; Table 1). At these loci, we also observed genome-wide significant associations with serum calcium, phosphate, PTH and ALP that do not in all cases correlate with the corresponding kidney stone association signal. Focusing on coding variants in genes with preferential renal expression, we also found two rare coding variants associating with kidney stones and recurrent stone formation.

### Variants at *ALPL*

**Figure 2:**
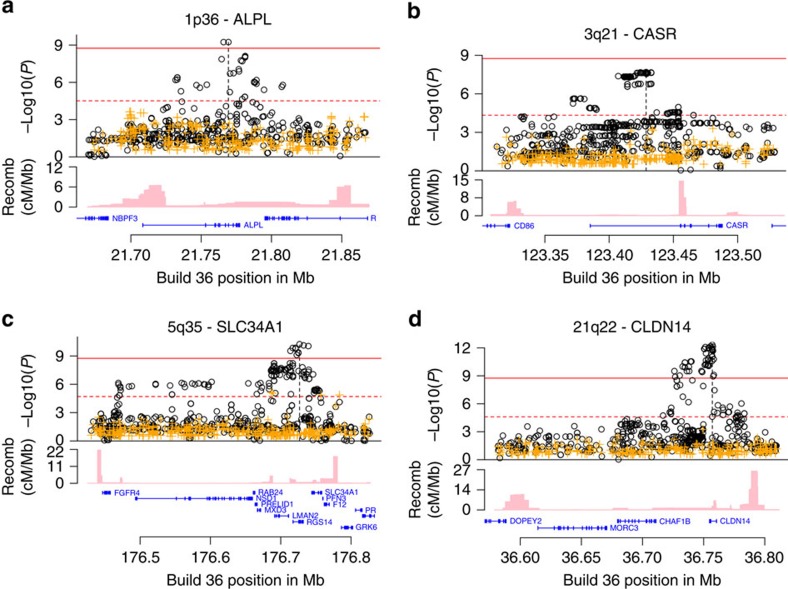
Locus plots corresponding to genome-wide significant and suggestive loci associating with kidney stone Logistic regression was used for association testing between sequence variants and disease, treating disease status as the response and genotype counts as covariates (**a**–**d**) For each plot, the −log10 *P* values, derived by logistic regression, (*y* axis) for the sequence variants are shown according to their chromosomal positions (*x* axis). The significant loci are at 1p36 (**a**), 5q35 (**c**), 21q22 (**d**) and suggestive locus at 3q21 (**b**). Orange sequence variant colour represent association values after adjusting for the leading variant. Estimated recombination rates are shown as pink lines and the genomic locations of genes at the locus are depicted in blue. The red solid line indicates the threshold for genome-wide statistical significance, which takes into account the effects of multiple testing (*P*=0.05/28.3 million=1.8 × 10^−9^). The red broken line indicates the significance threshold for claiming independence of the index varaint based on the number variants located within the LD block containing the index variant at each locus (ALPL; 0.05/1,625=3.1 × 10^−5^, CASR; 0.05/1,078=4.6 × 10^−5^, SLC34A1; 0.05/2,516=2.0 × 10^−5^, CLDN14; 0.05/1,993=2.5 × 10^−5^).

We observe a genome-wide significant signal with two fully correlated markers associating with kidney stones in ALPL at 1p36.12, represented by rs1256328 [T] (minor allele frequency (MAF)=17.79%, OR=1.21, *P*=5.8 × 10^−10^) (Fig. 2; Table 1; Supplementary Table 1). A missense variant in ALPL (rs34605986 [A], MAF=15.21%, NP_000469.3:p.Val522Ala) that correlates with rs1256328 (*r*^2^=0.73) also associates with kidney stones (OR=1.19, *P*=8.9 × 10^−8^) (Supplementary Table 1). When considering the 3,119 variants located within the linkage disequilibrium (LD) block containing rs1256328 no variants remained significant after adjusting for it (*P*>0.05/1,625=3.1 × 10^−5^) (Fig. 2; Supplementary Table 1). ALPL encodes a tissue nonspecific ALP. The sequence variant rs1256328 [T] and the correlated missense variant ALPL p.Val522Ala have a significant association with increased serum ALP levels in the general population (effect >7.8 s.d.%, *P*<2.2 × 10^−32^) (Table 2). To attempt replication, we directly genotyped the missense variant ALPL p.Val522Ala in a Danish population set (*N*=5,822, MAF_Denmark_=17.53%). We replicated the association (*P*=3.0 × 10^−4^) with an effect=10.73 s.d.%. Combined analysis of discovery and replication sets yielded a *P*=3.5 × 10^−35^ and an effect=8.00 s.d.% with no heterogeneity between the populations (*P*=0.38). This is consistent with a previous functional study where a mutated version of ALPL carrying p.Val522Ala showed increased enzyme activity in a cell-based assay^20^. Within ALPL, we also detect a low-frequency missense variant (rs149344982[A], MAF=1.42%, NP_000469.3:p.Arg152His), which associates strongly with reduced serum ALP levels (effect=−45.9 s.d.%, *P*=9.84 × 10^−105^) and suggestively with reduced risk of kidney stones (OR=0.742, *P*=8.1 × 10^−3^) (Supplementary Table 2). ALPL p.Arg152His has little correlation with the more common ALPL variants rs1256328 and p.Val522Ala (*r*^2^<0.0040) (Supplementary Table 3). This finding is in line with the effect of several rare loss-of-function variants in ALPL that have been reported in patients with hypophosphatasia (OMIM:146300), a syndrome characterized by decreased levels of ALP and elevated urine pyrophosphate 21, a known inhibitor of kidney stone formation^4^. ALPL is expressed in the proximal tubules of the kidney^21^ and hydrolyses pyrophosphate to free phosphate, where it may promote the formation of kidney stones^4^. Our results, for variants in ALPL that either increase or decrease ALP levels, suggest that the effect of ALPL on extracellular pyrophosphate metabolism can influence kidney stone formation.

The most significant association with serum phosphate levels in the Icelandic population is observed at the *ALPL* locus, represented by rs12132412[G] (MAF=34.85%, effect=5.0 s.d.%, *P*=1.8 × 10^−19^) (Supplementary Table 2). This variant remains significant (adjusted *P*=1.7 × 10^−6^, adjusted effect=3.9 s.d.%) after adjusting for the correlated rs1697421 (*r*^2^=0.54), which has been reported to associate with serum phosphate levels^22^. Notably, rs12132412[G] also associates with ALP levels (effect=−6.5 s.d.%, *P*=1.1 × 10^−34^) and is correlated (*r*^2^=0.41) with rs1976403, which has been associated with ALP levels^23^ (Supplementary Table 3). In the Icelandic population, the reported sequence variant rs1976403[C] associates significantly with both ALP (effect=10.4 s.d.%, *P*=4.3 × 10^−93^) and serum phosphate levels (effect=−3.7 s.d.%, *P* =5.2 × 10^−12^) (Supplementary Table 2). Interestingly, rs12132412 and rs1976403 have little correlation with rs1256328 and ALPL p.Arg152His that show association with kidney stones and demonstrate significant associasion with ALP at the *ALPL* locus (*r*^2^<0.0062) (Supplementary Table 2; Supplementary Table 4). Similarly, rs1256328 and ALPL p.Arg152His do not associate with serum phosphate levels (effect=−0.3 s.d.%, *P*=0.71 and effect=3.7 s.d.%, *P*=0.1, respectively) (Supplementary Table 2).

In summary, we report three uncorrelated (*r*^2^<0.0062 for all pairs) genome-wide significant signals at the *ALPL* locus associating with ALP and kidney stones (rs1256328 ), ALP and serum phosphate (rs12132412 and rs1976403) and ALP (*rs149344982*).

### Variants at *SLC34A1*

On 5q35.3 at the *SLC34A1* locus, encoding the Na/Pi co-transporter SLC34A1, we replicate with an effect size similar to the replicated one a common kidney stone association signal previously reported in an Asian population^14^ (Supplementary Table 5). The strongest marker in our data rs12654812[A] (MAF=41.84%) associates with kidney stones with an OR=1.18 and *P*=5.7 × 10^−11^ (Fig. 1; Table 1) and correlates with the previously reported marker (*r*^2^=0.68) (Supplementary Table 5). Nine variants located in or near *SLC34A1* show genome-wide significant association with kidney stones and correlate with the index variant rs12654812 (*r*^2^>0.63) (Fig. 2; Supplementary Table 6). In addition, we demonstrate that rs12654812[A] associates significantly with decreased serum PTH levels (effect=−5.9 s.d.%, *P*=2.3 × 10^−9^) and decreased serum phosphate (effect=−4.2 s.d.%, *P* =1.1 × 10^−14^) (Table 2). The sequence variant rs12654812 has also been reported by us and others to associate with kidney function-related traits^24^,^25^. To search for additional signals at this locus after adjusting for the index variant rs12654812, we performed conditional analysis including all variants located within the LD block containing rs12654812. We identified three strongly correlated (*r*^2^>0.90) rare variants, chr5:176686425[G], chr5:176688995[T] and chr5:176757439[G] (hg18) (MAF<0.50 %), that remained significant given the number of tests performed after adjusting for rs12654812 (*P*<2.0 × 10^−5^=0.05/2,516). One is a missense variant in *SLC34A1* (chr5:176757439[G] (NCBI36/hg18), NP_003043.3:p.Tyr489Cys, MAF=0.46 %) that was observed only once among 61,486 exomes in the Exome Aggregation Consortium (ExAC: http://exac.broadinstitute.org) database^26^ (*N* samples=2,535). SLC34A1 p.Tyr489Cys shows the best association of all coding variants (*N*=24) with kidney stone disease (adjusted OR=1.93, adjusted *P*=1.4 × 10^−5^) and recurrent stone formation (adjusted OR=2.58, adjusted *P*=4.3 × 10^−6^) (Table 3) in *SLC34A1* and the nine adjacent genes that reside within the same LD block (Fig. 2). The p.Tyr489Cys variant is located in the Na/Pi co-transporter domain^27^ (amino acids 368–504, see Supplementary Fig. 1) at a highly conserved position (GERP^28^=5.03) and is predicted to be pathogenic (PolyPhen^29^=probably damaging and SIFT^30^=deleterious). Interestingly, p.Tyr489Cys has been reported to associate with increased serum creatinine such as the common kidney stone risk variant rs12654812 [A]^25^. In our data, p.Tyr489Cys also associates with decreased PTH levels, similar to rs12654812[A] (Table 2; Supplementary Table 7).

The association data presented here point to *SLC34A1* as the kidney stone target at this locus. In support of this hypothesis, several studies demonstrate that rare variants in *SLC34A1* are linked to hypophosphatemic nephrolithiasis/osteoporosis (OMIM:612286) and that *SLC34A1* is the only gene at the locus that shows tissue-specific gene expression in kidney^31^ . Furthermore, the observed changes in biochemical traits reflect the function of *SLC34A1* as a phosphate transporter. The kidney is the main regulatory organ of phosphate homeostasis and serum phosphate levels are reflected by a change in phosphate reabsorption. *SLC34A1* is expressed in the brush border membrane of proximal tubular cells, where the bulk of phosphate reabsorption takes place, and appears to be responsible for ∼70% of total phosphate transport based on mouse models in which *Slc34a1* has been knocked out^32^,^33^,^34^. The reduction in serum PTH levels associated with the kidney stone variants likely results from a decrease in serum phosphate levels caused by diminished renal reabsorption as a consequence of the negative feedback loop that maintains serum phosphate levels.

### Variants at *CLDN14*

We previously reported a genome-wide significant association at the *CLDN14* locus on 21q22.13 with kidney stones represented by rs219780 (ref. 13). In the current analysis, a total of 31 correlated variants at the locus reach genome-wide significance, including six variants for recurrent kidney stones (Fig. 1; Fig. 2; Supplementary Table 8). The strongest signal associating with kidney stones is a two base pair deletion in intron 1 of *CLDN14* rs199565725[delAC] (MAF=23.68%, OR=0.81, *P*=4.7 × 10^−13^) (Table 1) correlating with the previously reported sequence variant rs219780[T] (*r*^2^=0.82, OR=0.81, *P*=2.6 × 10^−11^). We do not observe a significant residual signal when adjusting the association of rs199565725 with kidney stones for rs219780 (adjusted *P*=0.086). When considering the number of variants in the LD block containing rs199565725 no variants remained significant after adjusting for this variant (*P*>0.05/1,993=2.5 × 10^−5^).

*CLDN14* is a member of the claudin superfamily of proteins that regulate paracellular transport of ions and small solutes at epithelial tight junctions. We demonstrate that the sequence variant rs199565725[delAC] also associates with increased serum magnesium (effect=4.0 s.d.%, *P*=2.0 × 10^−7^), decreased serum potassium (effect=−1.2 s.d.%, *P*=8.2 × 10^−4^) and decreased PTH (effect=−4.5 s.d.%, *P*=1.1 × 10^−4^) (Table 2). Interestingly, *Cldn14* knockout mice have significantly higher serum magnesium levels than wild-type animals when kept on a high-calcium diet^35^. This might indicate that rs199565725[delAC] mediates a decrease of *CLDN14* gene function. Consistent with our previous study, rs199565725[delAC] shows a nominal association with bone mineral density (Supplementary Table 9).

### Variants at *CASR*

After the three genome-wide significant loci for kidney stones, we observe a suggestive association of rs7627468[A] in the first intron of *CASR* at 3q21.1 encoding the calcium-sensing receptor (MAF=26.80%; OR=1.16, *P*=2.0 × 10^−8^) (Table 1; Fig. 1). *CASR* is a G-protein-coupled receptor expressed on the apical surface of the proximal tubule^36^ that plays a key role in renal control of calcium homeostasis^37^ and has long been considered a candidate gene for kidney stones^38^,^39^. A total of 58 variants, strongly correlated with rs7627468 (*r*^2^>0.95) and located within intron 1 of *CASR*, associate with kidney stones (Fig. 2; Supplementary Table 10). A non-significant trend was observed for rs7627468[A] with increased serum calcium (effect=1.6 s.d.%, *P*=1.1 × 10^−3^), increased ionized calcium (effect=2.5 s.d.%, *P*=6.0 × 10^−3^) and with decreased 25-hydroxy vitamin D levels (effect=−4.6 s.d.%, *P*=7.5 × 10^−3^) (Table 2). We note that rs7627468 is not correlated with rs73186030, located 8-kb downstream of the *CASR* gene, (*r*^2^=0.0073) that is the most significant marker for serum calcium in the Icelandic population (effect=12.3 s.d.%, *P* =2.0 × 10^−61^) and is strongly correlated with rs1801725 (NP_000379.2:p.Ala986Ser) (*r*^2^=0.97), which has been associated with serum calcium^40^,^41^. The sequence variant rs73186030 does not associate with kidney stones (OR=0.96, *P*=0.33) and this association remains unaffected after adjusted for the kidney stone variant rs7627468 (Supplementary Table 11). Conversely, when adjusted for the serum calcium variant rs73186030, the association of the kidney stone variant rs7627468[A] with serum calcium (adjusted effect=2.2 s.d.%, adjusted *P*=3.9 × 10^−6^) and ionized serum calcium strengthens (adjusted effect=3.1 s.d.%, adjusted *P*=7.0 × 10^−4^) (Supplementary Table 11).

In summary, we observe two uncorrelated signals (*r*^2^=0.0073) at the *CASR* locus, one for serum calcium only (rs73186030) and one mainly for kidney stones (rs7627468). Taken together, this suggests that the effect of *CASR* on kidney stones is complex. The sequence variant rs73186030, which has a strong effect on serum calcium, does not associate with kidney stones. The kidney stone variant rs7627468 and other linked variants associated with kidney stones are located within intron 1 of *CASR* that entails a regulatory region^42^.

### Rare coding variant in *TRPV5*

**Figure 3:**
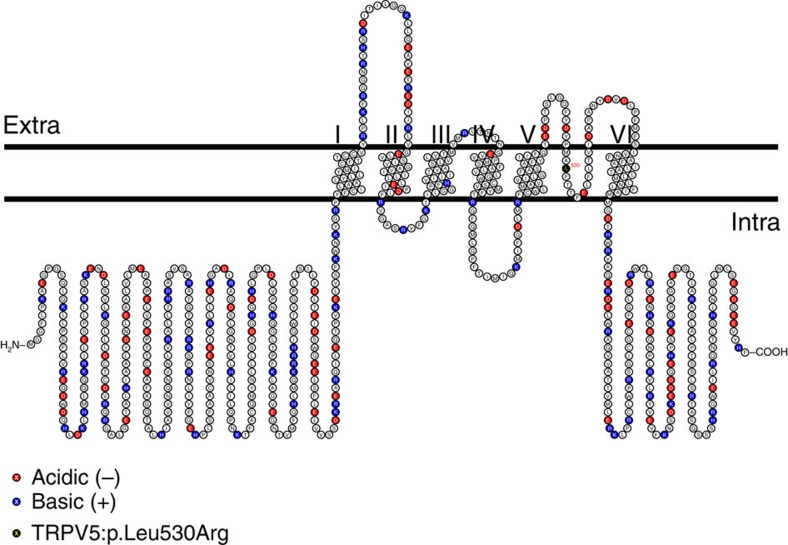
Schematic illustration of the topology of the TRPV5 protein The TRPV5 p.L530R mutation occurs within the pore-forming region (amino acids 527–538) of the ion transport domain (amino acids 389–578) of TRPV5 replacing a hydrophobic with a basic amino acid. Acidic and basic amino acids as red and blue, respectively.

We used a recent source of data on tissue-enriched gene expression in an attempt to analyse coding variants in genes with preferential kidney expression^31^. We tested a total of 220 coding variants in 63 genes, showing tissue-specific or enriched expression in the kidney, for association with kidney stones and recurrent kidney stones. In addition to SLC34A1 p.Tyr489Cys, we found a rare missense variant in *TRPV5* (NP_062815.2:p.Leu530Arg (MAF=0.13 %) associating significantly with recurrent kidney stones (OR=3.62, *P*=4.1 × 10^−5^<0.05/220=2.3 × 10^−4^) (Table 3). The TRPV5 p.Leu530Arg variant was observed only once in the ExAC database (samples *N*=61,486)^26^. The variant is at a highly conserved position (GERP^28^=5.8) and is predicted to be damaging by two different algorithms (PolyPhen^29^=probably damaging and SIFT^30^=deleterious). *TRPV5* is a highly selective epithelial calcium channel and the mutation lies within the pore-forming region of the protein (amino acids 527–538)^43^ (Fig. 3; Supplementary Fig. 1). The change in coding sequence results in substitution of the hydrophobic amino acid leucine with a positively charged arginine at position 530 (Fig. 3). The introduction of a positive charge into the hydrophobic pore-forming region of *TRPV5* is expected to interfere with the diffusion of positively charged calcium ions across the channel. *TRPV5* is expressed at the apical membrane of distal renal tubule epithelial cells that mediates calcium transport in the kidney and constitutes the rate-limiting step of active calcium reabsorption^44^. *Trpv5* knockout mice and mice carrying a point mutation in *Trpv5* exhibit renal calcium wasting resulting in severe hypercalciuria^45^,^46^. TRPV5 has been suggested to play a role in hypercalciuric disorders but candidate gene studies have so far failed to demonstrate an association^44^,^47^,^48^.

### Variant in *APRT* and recessive mode of inheritance

We note that we observe a strong association of a missense variant in the *APRT* gene encoding adenine phosphoribosyltransferase (rs104894506[A], NP_000476.1:p.Asp65Val, MAF=1.26%) with kidney stones under the recessive model (OR=31.97, *P*=6.83 × 10^−10^). This mutation has previously been reported to cause adenine phosphoribosyltransferase deficiency under a recessive mode of inheritance (OMIM:614723) with kidney stones as a hallmark clinical manifestation in Iceland^49^,^50^. Consistent with this observation, we do not observe a significant association of rs104894506[A] with kidney stones under the additive model (OR=1.01, *P*=0.93). No other variant showed a genome-wide significant association with kidney stones under the recessive model.

### Replication of Asian variants

A GWAS in Asians reported three loci associating with kidney stones^14^. In addition to the variants at the *SLC34A1* locus mentioned above, we were able to replicate variants at the *INMT-FAM188B-AQP1* locus (*P*<3.3 × 10^−3^, OR=1.16–1.21) (Supplementary Table 6).

## Discussion

We performed a GWAS of 28.3 million variants discovered through sequencing and observed four common sequence variants associating with the risk of kidney stones at *ALPL*, *SLC34A1*, *CLDN14* and *CASR*. We replicate the association of the variant at *SLC34A1* reported in Asians^14^ and we previously reported^13^ the signal corresponding to the association of the variant at *CLDN14*. In addition to the classic GWAS approach, we used a recent resource^31^ on tissue-specific expression together with the ability to detect and impute rare variants to analyse coding changes in genes with enriched expression in the kidney. Among those, we found rare missense variants at highly conserved sites in *SLC34A1* and *TRPV5* associating strongly with risk of kidney stones and recurrent kidney stones. The identification of a rare missense variant in *SLC34A1*, independent of common variants in the region, points to *SLC34A1* as the causative gene for both signals. Interestingly, the calcium channel *TRPV5* has been the focus of several studies suggesting a role in kidney stone formation^12^. Sequencing a large number of individuals (*N*=2,636) in a founder population allowed us to detect the TRPV5 p.Leu539Arg variant (MAF=0.13%). This variant is essentially absent from other sequenced populations and is located at an extremely conserved site in the pore-forming region of the protein, making it likely to be the causal variant explaining the association with recurrent kidney stones. The total proportion of sibling recurrence risk for kidney stones explained by the identified sequence variants is 4.81% (Supplementary Table 12).

Two of the identified genes are involved in phosphate homeostasis (ALPL^4^ and SLC34A1 (refs 12)) and the other three genes play a key role in renal handling of calcium (CLDN14 and CASR, TRPV5)^12^. We screened the kidney stones associated variants for their association to serum level of biochemical traits and detected association of variants at ALPL with ALP, SLC34A1 with PTH, and creatinine, phosphate and CLDN14 with PTH, magnesium and potassium. We also observed uncorrelated genome-wide significant association of variants at these loci that influence serum levels of biochemical traits but do not associate with the disease in all cases. This implies that the risk is not mediated solely through the serum levels of the biochemical traits.

The observation of three uncorrelated signals at *ALPL* associating with ALP levels which do not predict their associations with serum phosphate and kidney stones is noteworthy. This is an example of allelic heterogeneity that is particularly interesting in the context of the relationship between metabolic bone disease and kidney stones.

A similar pattern is observed at *CASR* where we observe two uncorrelated signals, one located in intron 1 of the *CASR* gene associating with kidney stones and the other at the 3′-end of the gene associating strongly with serum calcium but not with kidney stones. This observed allelic heterogeneity might reflect differences in the function of *CASR* in the kidney on one hand and the parathyroid gland on the other. Variations in intron 1 might specifically influence gene expression in the kidney influencing the ability of *CASR* to respond to extracellular calcium and in this way increase the risk of kidney stone formation^51^.

In summary, the genetic associations presented emphasize the role of sequence variants in genes involved in calcium–phosphate homeostasis in kidney stone disease. The pathophysiology underlying these associations requires further study.

## Methods

### The Icelandic study population

This study is based on whole-genome sequence data from the whole blood of 2,636 Icelanders participating in various disease projects at deCODE genetics^15^,^16^ (Supplementary Tables 13 and 14) (European Variant Archive: PRJEB8636). In addition, a total of 104,220 Icelanders have been genotyped using Illumina SNP chips^15^,^16^ (Supplementary Table 15) and genotype probabilites for untyped relatives has been calculated based on Icelandic genealogy^15^,^16^.

All participating individuals, or their guardians, gave their informed consent before blood samples were drawn. The family history of participants donating blood was incorporated into the study by including the phenotypes of first- and second-degree relatives and integrating over their possible genotypes.

All sample identifiers were encrypted in accordance with the regulations of the Icelandic Data Protection Authority. Approval for these studies was provided by the National Bioethics Committee and the Icelandic Data Protection Authority.

### Kidney stone study population

To identify kidney stone cases, we searched for patients with International Classification of Diseases (ICD) codes, radiology diagnosis codes and surgical procedure codes indicative of kidney stones^3^ at Landspitali—The National University Hospital of Iceland in Reykjavik (LUH), a community hospital for half of Iceland's population and a tertiary care centre for the whole nation; Akureyri Hospital in North Iceland, the largest hospital outside the Reykjavik area; and Domus Radiology in Reykjavik, the largest privately operated medical imaging clinic in the country. A thorough medical record review was conducted for all patients identified to confirm the diagnosis of kidney stones. Patients with calcifications other than kidney stones and asymptomatic kidney stones were excluded from the study. A total of 5,419 kidney stone cases were included in the association analysis; 2,979 of these were genotyped using various Illumina chips and imputed using long-range phased haplotypes, and genotype probabilities for 2,440 were imputed on the basis of information from genotyped close relatives^15^,^16^. Among the kidney stone cases were 2,172 recurrent kidney stone formers. A recurrent episode was defined as the development of a new stone occurring at least 6 months following the first stone event. Controls comprised individuals recruited through different genetic research projects at deCODE. Individuals in the kidney stone cohort were excluded from the control group. Of the controls, 88,266 were genotyped by chip, and 191,604 were imputed on the basis of the genotypes of close relatives^15^,^16^. The total number of controls was 279,870.

### Quantitative trait measurements

We studied a sample of Icelanders with biological markers measured at three different laboratories: the laboratory of the LUH, the Icelandic Medical Center (Laeknasetrid) Laboratory in Mjodd (RAM) and Akureyri Hospital (FSA). Here we use measurements of serum calcium (*N*=114,489, geometric mean (GM)=2.3), ionized serum calcium (*N*=18,516, GM=1.8), PTH (*N*=15,541, GM=2.0), 25-hydroxy vitamin D (*N*=7,544, GM=1.35), serum phosphate (*N*=51,056, GM=1.8), ALP (*N*=126,060, GM=2.6), bicarbonate (*N*=44,511, GM=1.7), chloride (*N*=92,938, GM=2.2), potassium (*N*=201,720, GM=4.2), sodium (*N*=198,119, GM=4.1) and uric acid (*N*=56,025, GM=1.9). The arithmetic mean of the quantile–quantile standardized trait values, adjusted for sex and age at the time of measurement for each individual, was used in the analysis.

A Danish sample set was included in the study involving measurement of ALP of healthy individuals from the Inter99 study^52^. The study was approved by the Regional Scientific Ethical Committees for Southern Denmark and the Capital Region of Denmark. Informed consent was obtained from all study participants.

### Gene and variant annotation

Variants were annotated with information from Ensembl release 70 using Variant Effect Predictor (VEP) version 2.8 (refs 52, 53).

### Association testing

To test for association between sequence variants and disease, we applied logistic regression using disease status as the response and genotype counts as covariates as we described previously^16^. The following individual characteristics that correlate with disease status were also included in the model as nuisance variables: sex, county of birth, current age or age at death (first- and second-order terms included), blood sample availability for the individual and an indicator function for the overlap of the lifetime of the individual with the timespan of phenotype collection.

As described previously^16^, given genotype counts for *n* individuals, 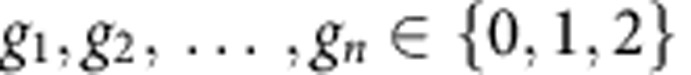, their phenotypes 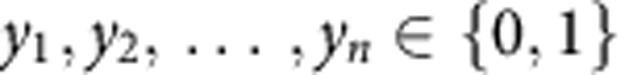 and a list of vectors of nuisance parameters 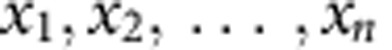, the logistic regression model states that


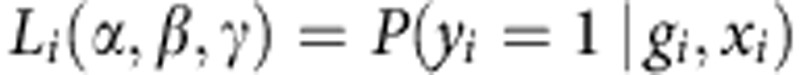



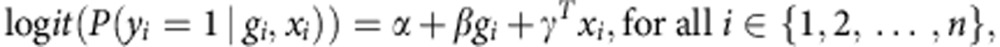


where *α*, *β* and *γ* are the regression coefficients and *L*_*i*_ is the contribution of the *i*th indivual to the likelihood function; 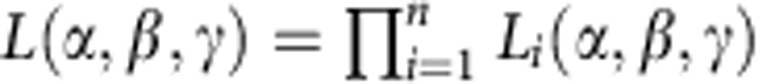. It is then possible to test for association based on the asymptotic assumption that the likelihood ratio statistic follows a *χ*^2^ distribution with one degree of freedom:


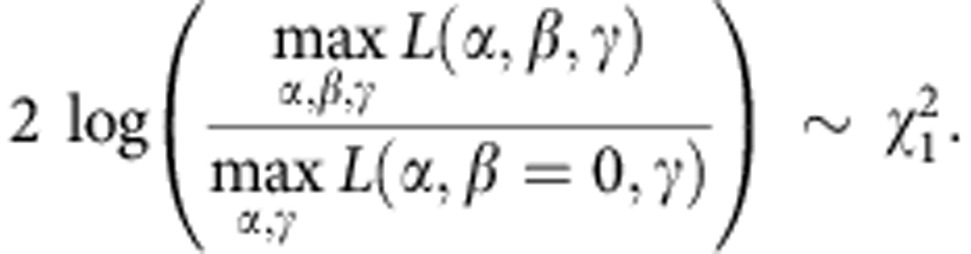


Given the computation cost of maximizing over the nuisance parameters for every marker in the genome, the likelihood was maximized under the null hypothesis of *β*=0, which is the same for all markers, and use the maximizer of *γ*, 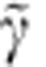, under the alternative. This methods leads to a smaller likelihood ratio than maximizing over *γ* for every marker because 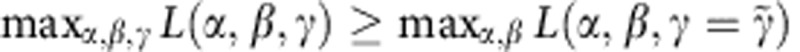.

Our analysis is based on imputed genotype values where the values of *g*_*i*_ are not known. Instead, we use *P*(*g*_*i*_=*j*|*I*_*i*_) for *j*∈{0,1,2}, where *I*_*i*_ stands for the information about *g*_*i*_. Given the logistic regression model above, this allows us to calculate


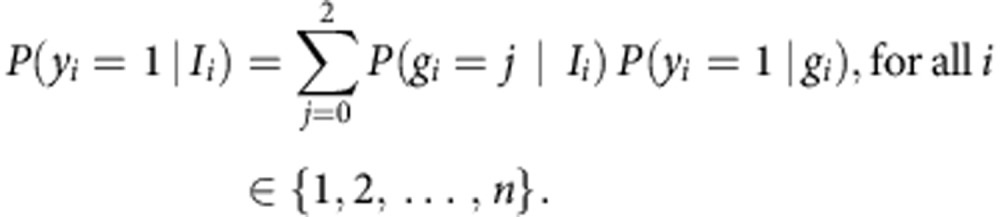


### Testing quantitative traits

To test for association between quantitative traits and sequence variants a generalized form of linear regression was used as described previously^16^. Let *y* be a vector of quantitative measurements, and let *g* be a vector of expected allele counts for the tested variant. Assuming the quantitative measurements follow a normal distribution with a mean that depends linearly on the expected allele at the variant and a variance covariance matrix proportional to the kinship matrix:


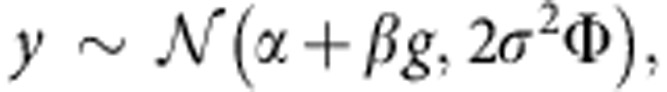


where


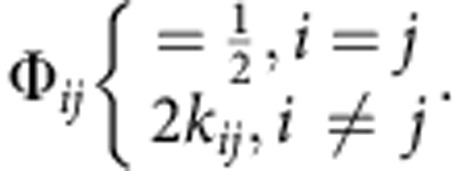


is the kinship matrix as estimated from the Icelandic genealogical database. We split individuals with trait values into smaller clusters for the calculation as it is not computationally feasible to use the full model. The maximum likelihood estimates for the parameters *α*, *β* and *σ* (ref. 2) involve inverting the kinship matrix. If there are *n* individuals in the cluster, then this inversion requires *O*(*n*^3^) calculations. However, because these calculations only need to be performed once, the computational cost of doing a genome-wide association scan will only be *O*(*n*^2^) calculations per variant, which is the cost of calculating the maximum likelihood estimates if the kinship matrix has already been inverted. The ALP measurements from the Danish Inter99 (ref. 54) study were tested using a generalized log-linear regression, assuming an additive genetic model, for association with allele count of rs34605986 using the R software package.

### Adjusting for relatedness

Relatedness and stratification within the sample sets were accounted for as we previously described^16^ using the genomic control method^55^. *P* values were adjusted by dividing the corresponding *χ*^2^ value with an inflation factor *λ*_g_ estimated based on a set of about 300,000 common variants distributed across the genome

### Inheritance models in association testing

The sum of the two imputed haplotype probabilities was used as a covariate for both the logistic regression and the generalized linear regression when testing for association under the additive model^16^. Where an individual was imputed to have the minor allele of a sequence variant with probability *a*_*f*_ on his paternal chromosome and *a*_*m*_ on his maternal chromosome, then *a*_*f*_+*a*_*m*_ was used as a covariate.

### Conditional analysis

When testing a variant conditioning on the expected genotypes of another variant the analysis was carried out using the same software used in the GWAS analysis^19^.

### Gene expression specificity classification

Genes were defined to have tissue-specific or tissue-enriched gene expression as described by Fagerberg *et al.*^31^(ArrayExpress: E-MTABB-1773). Briefly, tissue-specific expression is defined as at least 50-fold higher FPKM (fragments per kilobase of exon per million fragments mapped) than all other tissues (*N*=27) and tissue-enriched expression is defined as at least fivefold higher FPKM than all other tissues.

## Additional information

**How to cite this article:** Oddsson, A. *et al.* Common and rare variants associated with kidney stones and biochemical traits. *Nat. Commun.* 6:7975 doi: 10.1038/ncomms8975 (2015).

## Supplementary Material

Supplementary Information Supplementary Figure 1 and Supplementary Tables 1-15

## Figures and Tables

**Table 1:** Summary information for the lead regional sequence variants associating with kidney stone.

**SNP ID**	**Position (Hg18)**	**A**	**MAF (%) ice/1Keu**	**Locus**	**Kidney stones**		**Recurrent kidney stones**		**Mechanism**
					***P***	**OR**	***P***	**OR**	
rs199565725	chr21:36757108	A/AAC	23.68/23.20	*CLDN14*	4.7 × 10^−13^	0.81	3.5 × 10^−9^	0.77	Cell–cell adhesion
rs12654812	chr5:176726797	A/G	41.84/34.78	*SLC34A1*	5.7 × 10^−11^	1.18	4.4 × 10^−7^	1.21	Na/Pi co-transporter
rs1256328	chr1:21769354	T/C	17.79/17.32	*ALPL*	5.8 × 10^−10^	1.21	4.0 × 10^−6^	1.23	Alkaline phosphatase
rs7627468	chr3:123428789	A/G	26.80/24.02	*CASR*	2.0 × 10^−8^	1.16	4.1 × 10^−5^	1.18	Ca-sensing G-protein-coupled receptor

A, allele (minor/major); MAF, minor allele frequency (ice, Iceland; 1Keu, 1000 genomes European Americans); OR, odds ratio.

Reported are the three genome-wide significant (*P*<1.8 × 10^−9^) sequence variants (rs199565725, rs12654812 and rs1256328) and in addition one suggestive sequence variant (rs7627468) associating with kidney stone in Iceland.

**Table 2:** Kidney stone associated biochemical traits.

Trait*	**N (KS cases)**	***CLDN14*** **rs199565725[T]**		***SLC34A1*** **rs12654812[A]**		***ALPL*****rs1256328[T]**		***CASR*** **rs7627468[A]**	
		***P***	**Effect (s.d.%)**	***P***	**Effect (s.d.%)**	***P***	***Effect*****(s.d.%)**	***P***	**Effect (s.d.%)**
*Calcium–phosphate metabolism*									
** **ALP	126,060 (3,869)	0.30	−0.6	0.037	1.1	2.2 × 10^−32^	7.8	0.92	0.1
PTH	15,541 (1,003)	1.1 × 10^−4^	−4.5	2.3 × 10^−9^	−5.9	0.62	−0.6	0.13	1.7
25-OH VD	7,544 (377)	0.72	0.7	0.87	0.3	0.38	−1.7	7.5 × 10^−3^	−4.6
									
*Purine metabolism*									
Uric Acid	56,025 (2,667)	0.20	−1.0	0.090	1.1	0.56	−0.5	0.59	0.4
									
*Acid–base homeostasis*									
Bicarbonate	44,511 (1,576)	0.60	0.4	0.15	0.1	0.50	0.6	0.61	0.4
									
*Kidney function*									
Creatinine	195,933 (4,911)	0.17	−0.6	5.4 × 10^−8^	0.2	0.95	0.1	0.45	0.3
									
*Ion homeostasis*									
Calcium	114,489 (3,842)	0.34	0.5	0.012	1.1	0.79	−0.1	1.1 × 10^−3^	1.6
Calcium ionized	18,516 (1,129)	0.57	−0.5	0.026	1.8	0.63	0.5	6.0 × 10^−3^	2.5
Chloride	92,938 (3,228)	0.61	0.3	0.81	−0.1	0.09	−0.1	0.26	0.6
Magnesium	37,188 (1,472)	2.0 × 10^−7^	4.0	0.27	0.7	0.53	0.5	0.36	−0.7
Phosphate	51,056 (2,228)	0.11	1.0	1.1 × 10^−14^	−4.2	0.71	−0.3	0.01	−1.5
Potassium	201,720 (4,980)	8.2 × 10^−4^	−1.2	0.65	−0.1	0.80	0.1	0.05	0.7
Sodium	198,119 (4,951)	0.39	−0.3	0.019	0.8	0.41	0.3	0.11	−0.6

ALP, alkaline phosphatase; PTH, parathyroid hormone, 25-OH VD, 25-hydroxy vitamin D; *N*, number of individuals with genotype information with quantitative traits measurements

Association of genome-wide significant and suggestive kidney stone sequence variants with biochemical traits involved in calcium–phosphate metabolism, purine metabolism, kidney function, acid–base and ion homeostasis.

Underlined are *P* values reaching a significant threshold for the number of tests performed (0.05/52=9.6 × 10^−4^). The number of kidney stone cases in each group is indicated within brackets.

^*^All measurements are obtained from serum.

**Table 3:** Summary information for coding sequence variants in genes with specific or enriched expression in the kidney associating with kidney stone.

**Position (Hg18)**	**A**	**MAF (%)**	**Gene**	**HGVSp**	**Kidney stones**		**Recurrent kidney stones**		**Mechanism**
					P*	**OR**	P*	**OR**	
chr5:176757439	G/A	0.46	*SLC34A1*	NP_003043.3:p.Tyr489Cys	8.5 × 10^−5^	1.82	2.8 × 10^−5^	2.38	Na/Pi co-transporter
chr7:142319969	C/A	0.13	*TRPV5*	NP_062815.2:p.Leu530Arg	2.3 × 10^−3^	2.17	4.1 × 10^−5^	3.62	Ca channel

OR, odds ratio

^*^Genes with specific or enriched kidney expression: *N*=63. Coding variants with a MAF>0.01% within these genes: *N*=220, significance threshold for the number of test performed: 0.05/220=2.3 × 10^−4^.
