# Supplementary material for: Common and rare variants associated with kidney stones and biochemical traits
Source: Nat Commun. 2015 Aug 14;6:7975. doi: 10.1038/ncomms8975 (PMC4557269; doi:10.1038/ncomms8975)
Supplement: Supplementary Information — Supplementary Figure 1 and Supplementary Tables 1-15 [file ncomms8975-s1.pdf]

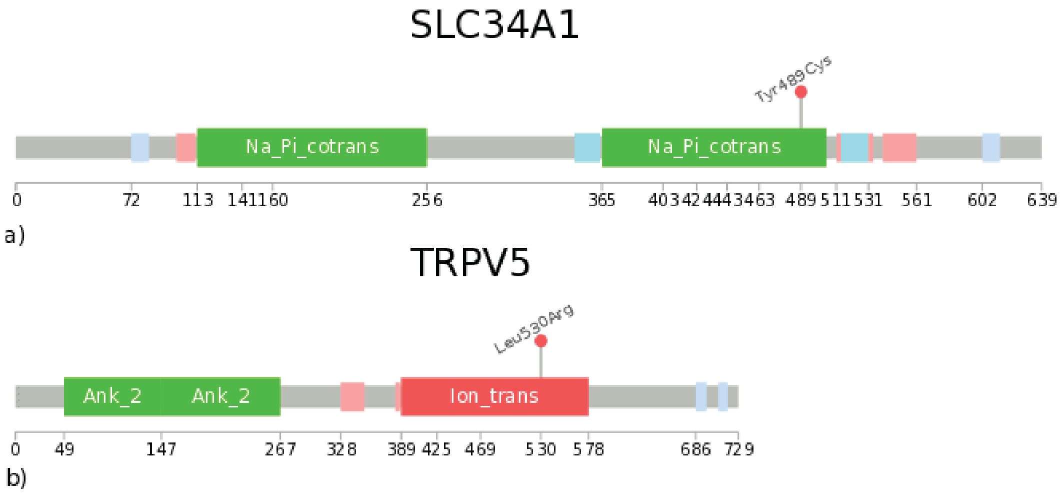

Supplementary Figure 1 A lollipop plot illustrating the SLC34A1 Tyr489Cys a) and TRPV5 Leu530Arg b) mutations including an illustrative domain graphic of protein sequence features. Pfam Regions/domains for transmembrane and low-complexity regions are shown as rectangles in the colors pink and blue, respectively. Pfam A domains are shown as rectangles in the colors green or red. The amino acid positions are plotted horizontally on the x-axis.

Supplementary Table 1 Conditional analysis of genome-wide significant association signals with kidney stones at the ALPL locus by conditioning on rs1256328

					Kidney Stones				Recurrent Kidney stones		
Position (Hg18)	SNP ID	minA	majA	MAF	Unadjusted P	Unadjusted OR	Adjusted P	Adjusted OR	*P*	OR	r^2
chr1:21769354	rs1256328	T	C	17.79	$5.8x10^{-10}$	1.21	-	-	$4.0×10^{-6}$	1.23	-
chr1:21765931	rs1256332	A	C	17.66	$5.8x10^{-10}$	1.21	0.96	0.98	$3.3×10^{-6}$	1.24	0.99
chr1:21776718	rs34605986	C	T	15.21	$8.9x10^{-8}$	1.19	0.87	0.99	$2.8×10^{-3}$	1.19	0.73

Supplementary Table 2 Conditional association analysis of the variants rs1256328, rs149344982, rs12132412 and rs1976403 at the *ALPL* locus for kidney stones, alkaline phosphatase and serum phosphate.

				Unadjusted		Adjusted - rs1256328		Adjusted - rs149344982		Adjusted - rs12132412		Adjusted - rs1976403	
	Position(Hg18)	SNP ID	MAF(%)	OR/ Effect(SD%)	*P*	OR/ Effect(SD%)	*P*	OR/ Effect(SD%)	*P*	OR/ Effect(SD%)	*P*	OR/ Effect(SD%)	*P*
**Kidney Stones**	chr1:21769354	rs1256328†	17.79	1.21	$5.8x10^{-10}$	-	-	1.21	$1.3x10^{-9}$	1.21	$4.6x10^{-10}$	1.21	$6.4x10^{-10}$
	chr1:21762347	rs149344982‡	1.42	0.74	$8.1x10^{-3}$	0.78	0.028	-	-	0.74	$6.8x10^{-3}$	0.75	$9.2x10^{-3}$
	chr1:21692629	rs12132412	34.85	0.97	0.26	0.95	0.040	0.95	0.064	-	-	0.96	0.23
	chr1:21639040	rs1976403	44.80	1.02	0.32	1.01	0.70	1.02	0.49			-	-
**Alkaline Phosphatase**	chr1:21769354	rs1256328†	17.79	7.8	$2.2x10^{-32}$	-	-	7.0	$1.9x10^{-26}$	8.1	$2.3x10^{-34}$	6.9	$3.1x10^{-25}$
	chr1:21762347	rs149344982‡	1.42	-45.9	$9.5x10^{-105}$	-44.6	$7.9x10^{-99}$	-	-	-47.7	$1.3x10^{-112}$	-42.7	$1.3x10^{-90}$
	chr1:21692629	rs12132412	34.85	-6.5	$1.1×10^{-34}$	-6.7	$1.1x10^{-36}$	-7.2	$1.3x10^{-42}$	-	-	0.7	0.32
	chr1:21639040	rs1976403	44.80	10.4	$4.3×10^{-93}$	10.0	$5.6x10^{-86}$	9.6	$8.5x10^{-79}$	10.8	$2.0×10^{-60}$	-	-
**Serum Phosphate**	chr1:21769354	rs1256328†	17.79	-0.3	0.71	-	-	-0.2	0.78	-0.4	0.55	0.1	0.86
	chr1:21762347	rs149344982‡	1.42	3.7	0.10	3.7	0.10	-	-	4.9	0.031	2.5	0.27
	chr1:21692629	rs12132412	34.85	5.0	$1.8×10^{-19}$	5.0	$1.6×10^{-19}$	5.1	$6.7x10^{-20}$	-	-	4.3	$1.8×10^{-9}$
	chr1:21639040	rs1976403	44.80	-3.7	$5.2×10^{-12}$	-3.7	$5.5x10^{-12}$	-3.6	$1.1x10^{-11}$	-1.0	0.13	-	-

†NP_000469.3:p.Val522Ala
‡NP_000469.3:p.Arg152His

Supplementary Table 3 Pattern of linkage disequilibrium for the variants rs1256328, rs149344982, rs12132412 and rs1976403 at the *ALPL* locus.

			rs1256328			rs149344982			rs12132412			rs1976403		
Position(Hg18)	SNP ID	MAF(%)	r^2	D'	Sign	r^2	D'	Sign	r^2	D'	Sign	r^2	D'	sign
chr1:21769354	rs1256328	17.79	-	-		0.0032	1.0	-1	0.0011	0.053	1	0.0050	0.14	1
chr1:21762347	rs149344982	1.42	0.0032	1.00	-1	-	-		0.0039	0.72	-1	0.0061	0.73	-1
chr1:21692629	rs12132412	34.85	0.0011	0.053	1	0.0039	0.72	-1	-	-		0.41	0.97	-1

Supplementary Table 4 Conditional association analysis of the variants rs12132412 and rs1697421 at the *ALPL* locus for serum phosphate.

Position(Hg18)	SNP ID	MAF(%)	Covariate SNP ID	Unadjusted Effect (SD%)	Unadjusted *P*	Adjusted Effect (SD%)	Adjusted *P*	Phenotype
chr1:21692629	rs12132412†	34.85	rs1697421	5.0	1.8×10^{-19}	3.9	1.7×10^{-6}	Phosphate
chr1:21695879	rs1697421†	49.77	rs12132412	4.2	3.1×10^{-15}	1.5	0.055	Phosphate

†R^2=0.54

Supplementary Table 5 Sequence variants previously reported to associate with kidney stones in the Japanese population by Urabe *et al*

					Japan*		Iceland			Best association in Iceland with kidney stones among variants correlated (R^2>0.8) in Asians					
Locus	Position(Hg18)	SNP ID	RA	RAF(%)	OR	*P*	OR	*P*	# r^2 > 0.8 in CHBJPT	SNP ID	RA	Position(Hg18)	OR	*P*	r^2 chbjpt/Ice
**SLC34A1**	chr5:176730912	rs11746443**	A	34.74	1.19	8.5×10^{-12}	1.16	1.6×10^{-8}	6	rs4075958	A	chr5:176717118	1.16	6.0×10^{-9}	0.96/0.88
	chr5:176726797	rs12654812**	A	41.84	1.16	4.4×10^{-11}	1.18	5.7×10^{-11}	3	-	-	-	-	-	-
**INMT-FAM188B-AQP1**	chr7:30903703	rs1000597†	C	8.35	1.22	2.2×10^{-14}	1.16	4.7×10^{-4}	3	-	-	-	-	-	-
	chr7:30882003	rs12669187†	A	3.46	1.25	1.5×10^{-12}	1.21	3.3×10^{-3}	1	-	-	-	-	-	-
**DGKH**	chr13:41600711	rs1170155‡	C	32.81	1.15	3.9×10^{-9}	1.00	0.96	3	rs9533007	A	chr13:41608235	0.99	0.63	0.93/0.35
	chr13:41652522	rs4142110‡	C	45.42	1.14	4.6×10^{-9}	1.07	0.010	6	rs12585865	C	chr13:41649707	1.07	9.6×10^{-3}	0.85/0.99
	chr13:41588060	rs7981733‡	C	53.19	1.14	1.4×10^{-8}	1.02	0.41	5	-	-	-	-	-	-

*PMID: 22396660, RA: Risk allele, RAF: risk allele frequency (Iceland)
**R^2= 0.68
†R^2= 0.39
‡R^2 rs1170155/rs4142110; R^2 = 0.056, rs1170155/rs7981733; R^2 = 0.42, rs4142110/rs7981733; R^2 = 0.40

Supplementary Table 6 Conditional analysis of genome-wide significant association signals at the *RGS14-SLC34A1* locus by conditioning on rs12654812.

					Kidney Stones				Recurrent Kidney Stones		
Position (Hg18)	SNP ID	minA	majA	MAF	Unadjusted P	Unadjusted OR	Adjusted P	Adjusted OR	*P*	OR	r^2
chr5:176726797	rs12654812	A	G	41.84	5.7x10-11	1.18	-	-	4.4×10^{-7}	1.21	-
chr5:176732598	rs10051765	C	T	39.31	7.6x10-11	1.18	0.21	1.09	2.6×10^{-6}	1.19	0.83
chr5:176739242	rs35716097	T	C	33.57	9.0x10-11	1.18	0.050	1.09	8.7×10^{-7}	1.21	0.64
chr5:176721228	rs4976647	C	A	39.83	1.4x10-10	1.17	0.57	1.05	1.4×10^{-7}	1.22	0.89
chr5:176717054	rs4976689	G	C	39.84	1.4x10-10	1.17	0.56	1.05	8.6×10^{-8}	1.22	0.89
chr5:176721176	rs4976646	C	T	39.91	1.8x10-10	1.17	0.54	1.05	1.2×10^{-7}	1.22	0.89
chr5:176713815	rs7713145	A	G	37.73	2.9x10-10	1.17	0.068	1.09	3.9×10^{-5}	1.17	0.63
chr5:176725097	rs6556313	G	A	40.61	4.3x10-10	1.17	0.73	0.96	2.4×10^{-7}	1.21	0.92
chr5:176725163	rs11135015	C	T	40.56	4.9x10-10	1.17	0.72	0.96	3.9×10^{-7}	1.21	0.92
chr5:176722768	rs67111717	G	A	39.73	7.8x10-10	1.17	0.96	1.00	$1.0s\times10^{-7}$	1.22	0.88

Supplementary Table 7 Association of coding sequence variants in genes with specific or enriched gene expression in the kidney biochemical traits involved in calcium-phosphate metabolism, purine metabolism, kidney function, acid-base and ion homeostasis.

			SLC34A1 chr5:176757439:S		TRPV5 chr7:142319969:S	
	Trait[†]	N (KS cases)	P-value	Effect(SD%)	P-value	Effect(SD%)
Calcium-Phosphate Metabolism	ALP	126,060 (3,869)	0.045	-7.7	0.11	11.0
	PTH	15,541 (1,003)	2.5×10^{-4}	-25.6	0.01	27.9
	25-OH VD	7,544 (377)	0.43	8.6	0.83	3.5
Purine Metabolism	Uric Acid	56,025 (2,667)	0.48	-3.5	0.54	-5.1
Acid-Base Homeostasis	Bicarbonate	44,511 (1,576)	0.32	5.2	0.96	-0.4
Kidney Function	Creatinine	195,933 (4,911)	8.4×10^{-6}	12.1	0.05	9.6
Ion Homeostasis	Calcium	114,489 (3,842)	0.044	6.5	0.09	-9.5
	Calcium Ionized	18,516 (1,129)	0.015	14.9	0.05	-21.0
	Chloride	92,938 (3,228)	0.015	8.3	0.97	0.2
	Magnesium	37,188 (1,472)	0.16	6.7	0.53	5.1
	Phosphate	51,056 (2,228)	0.48	-16.3	0.11	-10.3
	Potassium	201,720 (4,980)	0.83	0.5	0.28	4.6
	Sodium	198,119 (4,951)	0.41	2.0	0.12	-6.8

Underlined are P-values that reach a significant threshold for the number of biochemical traits tested ($0.05/26 = 1.9\text{x } 10^{-3}$).). ALP = alkaline phosphatase, PTH = parathyroid hormone, 25-OH VD = 25-hydroxy vitamin D
†All measurements are obtained from serum.

Supplementary Table 8 Conditional analysis of genome-wide significant association signals for kidney stones at the *CLDN14* locus by conditioning on rs199565725.

					Kidney Stones				Recurrent Kidney Stones		
Position (Hg18)	SNP ID	minA	majA	MAF	Unadjusted P	Unadjusted OR	Adjusted P	Adjusted OR	*P*	OR	r^2
chr21:36757108	rs199565725	A	AAC	23.70	$4.7\text{x}10^{-13}$	0.81	-	-	$3.5\text{x}10^{-9}$	0.77	-
chr21:36755621	rs219779	A	G	24.39	$6.7\text{x}10^{-13}$	0.81	0.90	0.98	$5.8\text{x}10^{-10}$	0.76	0.96
chr21:36757518	rs219770	G	A	24.44	$7.5\text{x}10^{-13}$	0.81	0.76	1.06	$6.7\text{x}10^{-10}$	0.76	0.96
chr21:36751378	rs219787	T	C	24.54	$8.2\text{x}10^{-13}$	0.81	0.99	1.00	$1.3\text{x}10^{-9}$	0.76	0.95
chr21:36756511	rs219778	G	A	24.57	$9.6\text{x}10^{-13}$	0.81	0.96	1.01	$2.1\text{x}10^{-9}$	0.77	0.95
chr21:36754491	rs219781	T	G	24.58	$1.0\text{x}10^{-12}$	0.81	1.00	1.00	$2.0\text{x}10^{-9}$	0.77	0.95
chr21:36754278	rs219782	G	A	24.56	$1.1\text{x}10^{-12}$	0.81	1.00	1.00	$2.1\text{x}10^{-9}$	0.77	0.95
chr21:36750428	rs219791	G	T	25.83	$1.3\text{x}10^{-12}$	0.81	0.62	0.96	$1.0\text{x}10^{-9}$	0.77	0.85
chr21:36756705	rs219776	C	T	24.54	$1.3\text{x}10^{-12}$	0.81	0.72	1.07	$1.9\text{x}10^{-9}$	0.77	0.96
chr21:36750732	rs219790	G	T	26.03	$1.3\text{x}10^{-12}$	0.82	0.92	0.99	$6.1\text{x}10^{-10}$	0.77	0.88
chr21:36757203	rs219773	A	G	24.54	$1.5\text{x}10^{-12}$	0.81	0.72	1.07	$2.4\text{x}10^{-9}$	0.77	0.96
chr21:36756814	rs219775	G	A	24.55	$1.6\text{x}10^{-12}$	0.81	0.72	1.07	$2.7\text{x}10^{-9}$	0.77	0.96
chr21:36757217	rs219772	T	A	24.54	$1.6\text{x}10^{-12}$	0.81	0.72	1.07	$2.6\text{x}10^{-9}$	0.77	0.96
chr21:36756784	rs2835363	T	A	24.52	$1.6\text{x}10^{-12}$	0.81	0.72	1.07	$2.3\text{x}10^{-9}$	0.77	0.96
chr21:36757371	rs219771	T	C	24.55	$1.7\text{x}10^{-12}$	0.81	0.72	1.07	$2.7\text{x}10^{-9}$	0.77	0.96
chr21:36757545	rs219769	A	C	24.56	$1.8\text{x}10^{-12}$	0.81	0.72	1.07	$2.6\text{x}10^{-9}$	0.77	0.96
chr21:36757634	rs219768	G	C	24.54	$2.1\text{x}10^{-12}$	0.81	0.72	1.07	$2.8\text{x}10^{-9}$	0.77	0.96
chr21:36756591	rs219777	A	G	23.48	$5.2\text{x}10^{-12}$	0.81	0.70	0.91	$6.2\text{x}10^{-9}$	0.77	0.97
chr21:36753822	rs219783	G	C	23.61	$1.5\text{x}10^{-11}$	0.82	0.66	0.91	$2.5\text{x}10^{-8}$	0.78	0.96
chr21:36753737	rs219784	T	C	23.62	$1.5\text{x}10^{-11}$	0.82	0.73	0.93	$2.5\text{x}10^{-8}$	0.78	0.96
chr21:36758248	rs219767	T	C	23.40	$1.6\text{x}10^{-11}$	0.82	0.64	0.92	$1.5\text{x}10^{-8}$	0.77	0.95
chr21:36753621	rs219786	C	T	20.73	$2.1\text{x}10^{-11}$	0.81	0.42	0.94	$3.7\text{x}10^{-9}$	0.76	0.82
chr21:36756913	rs219774	T	C	23.59	$2.6\text{x}10^{-11}$	0.82	0.97	0.99	$3.4\text{x}10^{-8}$	0.78	0.96
chr21:36755177	rs219780	T	C	20.80	$2.6\text{x}10^{-11}$	0.81	0.43	0.94	$4.4\text{x}10^{-9}$	0.76	0.82
chr21:36751875	rs2850087	A	T	23.21	$5.9\text{x}10^{-11}$	0.82	0.99	1.00	$7.3\text{x}10^{-10}$	0.75	0.01
chr21:36753730	rs219785	C	G	20.87	$6.3\text{x}10^{-11}$	0.81	0.44	0.94	$1.6\text{x}10^{-8}$	0.77	0.81
chr21:36751032	rs219788	C	T	20.87	$6.8\text{x}10^{-11}$	0.81	0.44	0.94	$1.3\text{x}10^{-8}$	0.76	0.81
chr21:36750910	rs219789	A	G	20.87	$6.9\text{x}10^{-11}$	0.81	0.45	0.94	$1.3\text{x}10^{-8}$	0.76	0.81
chr21:36748180	rs219793	T	C	20.85	$3.1\text{x}10^{-10}$	0.82	0.57	0.96	$3.8\text{x}10^{-8}$	0.77	0.79
chr21:36758347	rs219766	G	C	25.02	$8.1\text{x}10^{-10}$	0.84	0.50	1.05	$2.2\text{x}10^{-7}$	0.80	0.82
chr21:36757852	rs12626330	G	C	48.09	$1.2\text{x}10^{-9}$	1.16	0.015	1.07	$3.5\text{x}10^{-8}$	1.22	0.29

Supplementary Table 9 Association of kidney stone associated sequence variants with whole body bone mineral desnity (N = 7,756).

SNP ID	Position (Hg18)	minA/majA	MAF	Locus	Effect	*P*
chr21:36757108:I	chr21:36757108	A/AAC	23.70	CLDN14	0.074	6.3×10^{-4}
rs12654812	chr5:176726797	A/G	41.84	RGS14	-0.016	0.39
rs1256328	chr1:21769354	T/C	17.79	ALPL	-0.018	0.44
chr7:142319969:S	chr7:142319969	C/A	0.13	TRPV5	-0.07	0.74
rs7627468	chr3:123428789	A/G	26.80	CASR	0.006	0.77
chr5:176757439:S	chr5:176757439	G/A	0.46	SLC34A1	-0.034	0.82

MAF = minor allele frequency (Iceland), mina = minor allele, majA = major allele

Supplementary Table 10 Conditional analysis of genome-wide significant association signals with kidney stones at the *CASR* locus by conditioning on rs7627468.

					Kidney Stones				Recurrent Kidney Stones		
**Position (Hg18)**	**SNP ID**	**minA**	**majA**	**MAF**	**Unadjusted P**	**Unadjusted OR**	**Adjusted P**	**Adjusted OR**	***P***	**OR**	**r^2**
chr3:123428789	rs7627468	A	G	26.80	$2.0x10^{-8}$	1.16	-	-	$4.1x10^{-5}$	1.18	-
chr3:123424375	chr3:123424375:I	T	TA	26.97	$2.1x10^{-8}$	1.16	1.00	1.00	$3.3x10^{-5}$	1.18	0.99
chr3:123432660	rs7639847	A	G	26.94	$2.2x10^{-8}$	1.16	1.00	1.00	$4.0x10^{-5}$	1.18	0.99
chr3:123432755	rs7629603	T	C	26.93	$2.3x10^{-8}$	1.16	1.00	1.00	$4.2x10^{-5}$	1.18	0.99
chr3:123432758	rs7617898	C	G	26.93	$2.3x10^{-8}$	1.16	1.00	1.00	$3.8x10^{-5}$	1.18	0.99
chr3:123429066	rs4678187	T	C	26.94	$2.3x10^{-8}$	1.16	1.00	1.00	$4.3x10^{-5}$	1.18	0.99
chr3:123427039	rs2332237	C	A	26.89	$2.3x10^{-8}$	1.16	1.00	1.00	$4.5x10^{-5}$	1.18	0.99
chr3:123424146	rs1354159	C	T	26.92	$2.3x10^{-8}$	1.16	1.00	1.00	$4.0x10^{-5}$	1.18	0.99
chr3:123424992	rs9821555	T	G	26.92	$2.3x10^{-8}$	1.16	1.00	1.00	$4.0x10^{-5}$	1.18	0.99
chr3:123421593	rs1501892	G	A	26.92	$2.4x10^{-8}$	1.16	1.00	1.00	$4.0x10^{-5}$	1.18	0.99
chr3:123425502	rs2202126	A	G	26.92	$2.4x10^{-8}$	1.16	1.00	1.00	$4.1x10^{-5}$	1.18	0.99
chr3:123426549	rs61203229	A	G	26.92	$2.4x10^{-8}$	1.16	1.00	1.00	$4.0x10^{-5}$	1.18	0.99
chr3:123428160	rs6767350	T	C	26.92	$2.4x10^{-8}$	1.16	1.00	1.00	$4.2x10^{-5}$	1.18	0.99
chr3:123425403	chr3:123425403:I	G	GA	26.92	$2.4x10^{-8}$	1.16	1.00	1.00	$4.0x10^{-5}$	1.18	0.99
chr3:123422665	rs4303826	G	A	26.92	$2.4x10^{-8}$	1.16	1.00	1.00	$4.0x10^{-5}$	1.18	0.99
chr3:123428528	rs11918240	A	G	26.92	$2.4x10^{-8}$	1.16	1.00	1.00	$4.2x10^{-5}$	1.18	0.99
chr3:123429991	rs9875636	G	A	26.93	$2.4x10^{-8}$	1.16	1.00	1.00	$4.3x10^{-5}$	1.18	0.99
chr3:123429428	rs55708281	A	T	26.92	$2.4x10^{-8}$	1.16	1.00	1.00	$4.2x10^{-5}$	1.18	0.99
chr3:123430215	rs4678172	T	G	26.93	$2.4x10^{-8}$	1.16	1.00	1.00	$4.3x10^{-5}$	1.18	0.99
chr3:123429455	rs56242285	A	C	26.93	$2.4x10^{-8}$	1.16	1.00	1.00	$4.2x10^{-5}$	1.18	0.99
chr3:123429490	chr3:123429490:I	C	CTG	26.93	$2.4x10^{-8}$	1.16	1.00	1.00	$4.2x10^{-5}$	1.18	0.99
chr3:123427854	rs6767061	T	C	26.92	$2.4x10^{-8}$	1.16	1.00	1.00	$4.2x10^{-5}$	1.18	0.99
chr3:123429045	rs4678188	T	C	26.97	$2.8x10^{-8}$	1.16	1.00	1.00	$4.3x10^{-5}$	1.18	0.99
chr3:123420153	chr3:123420153:I	GAAG	G	26.50	$3.9x10^{-8}$	1.16	0.67	0.93	$2.4x10^{-5}$	1.19	0.97
chr3:123420151	chr3:123420151:I	TAGA	T	26.50	$3.9x10^{-8}$	1.16	0.67	0.93	$2.4x10^{-5}$	1.19	0.97
chr3:123407074	rs7614486	G	T	26.64	$4.2x10^{-8}$	1.16	0.65	0.93	$1.4x10^{-5}$	1.19	0.96
chr3:123416195	rs6438712	A	G	26.58	$4.4x10^{-8}$	1.16	0.59	0.91	$2.3x10^{-5}$	1.19	0.96
chr3:123418957	rs11721042	A	G	26.59	$4.5x10^{-8}$	1.16	0.59	0.91	$2.4x10^{-5}$	1.19	0.96
chr3:123418791	rs11720986	A	G	26.59	$4.5x10^{-8}$	1.16	0.59	0.91	$2.4x10^{-5}$	1.19	0.96
chr3:123418968	rs11717321	T	C	26.59	$4.5x10^{-8}$	1.16	0.59	0.91	$2.4x10^{-5}$	1.19	0.96
chr3:123418890	rs11713215	G	A	26.59	$4.5x10^{-8}$	1.16	0.59	0.91	$2.4x10^{-5}$	1.19	0.96
chr3:123418516	rs3845917	G	A	26.59	$4.6x10^{-8}$	1.16	0.59	0.91	$2.5x10^{-5}$	1.19	0.96
chr3:123413461	rs6788470	G	A	26.58	$4.6x10^{-8}$	1.16	0.59	0.91	$2.4x10^{-5}$	1.19	0.96
chr3:123418797	rs11713180	G	A	26.59	$4.6x10^{-8}$	1.16	0.59	0.91	$2.5x10^{-5}$	1.19	0.96
chr3:123411372	rs7638770	T	C	26.58	$4.6x10^{-8}$	1.16	0.59	0.91	$2.4x10^{-5}$	1.19	0.96
chr3:123411897	rs12489909	G	T	26.58	$4.7x10^{-8}$	1.16	0.59	0.91	$2.5x10^{-5}$	1.19	0.96
chr3:123412376	rs12490055	G	A	26.58	$4.7x10^{-8}$	1.16	0.59	0.91	$2.5x10^{-5}$	1.19	0.96
chr3:123413340	chr3:123413340:I	GA	G	26.58	$4.7x10^{-8}$	1.16	0.59	0.91	$2.5x10^{-5}$	1.19	0.96
chr3:123412140	rs12496336	A	G	26.58	$4.7x10^{-8}$	1.16	0.59	0.91	$2.5x10^{-5}$	1.19	0.96
chr3:123414399	rs11914420	A	G	26.58	$4.7x10^{-8}$	1.16	0.59	0.91	$2.4x10^{-5}$	1.19	0.96
chr3:123414085	rs4678124	A	C	26.57	$4.7x10^{-8}$	1.16	0.66	0.93	$2.5x10^{-5}$	1.19	0.96
chr3:123412996	rs6763316	A	G	26.58	$4.7x10^{-8}$	1.16	0.59	0.91	$2.5x10^{-5}$	1.19	0.96
chr3:123413478	rs6763821	A	G	26.58	$4.7x10^{-8}$	1.16	0.59	0.91	$2.5x10^{-5}$	1.19	0.96
chr3:123413210	rs6787967	C	T	26.58	$4.7x10^{-8}$	1.16	0.59	0.91	$2.5x10^{-5}$	1.19	0.96
chr3:123413689	rs4678123	C	T	26.59	$4.7x10^{-8}$	1.16	0.59	0.91	$2.5x10^{-5}$	1.19	0.96
chr3:123416325	rs6438713	C	T	26.59	$4.7x10^{-8}$	1.16	0.59	0.91	$2.4x10^{-5}$	1.19	0.96
chr3:123413530	rs6788304	C	A	26.59	$4.7x10^{-8}$	1.16	0.59	0.91	$2.5x10^{-5}$	1.19	0.96
chr3:123414887	rs6776376	G	C	26.59	$4.7x10^{-8}$	1.16	0.59	0.91	$2.5x10^{-5}$	1.19	0.96
chr3:123416430	chr3:123416430:I	CT	C	26.59	$4.7x10^{-8}$	1.16	0.59	0.91	$2.4x10^{-5}$	1.19	0.96
chr3:123415756	rs7647612	A	G	26.58	$4.7x10^{-8}$	1.16	0.59	0.91	$2.4x10^{-5}$	1.19	0.96
chr3:123414782	rs6776280	G	A	26.59	$4.7x10^{-8}$	1.16	0.59	0.91	$2.4x10^{-5}$	1.19	0.96
chr3:123416523	rs9820206	A	G	26.59	$4.7x10^{-8}$	1.16	0.59	0.91	$2.5x10^{-5}$	1.19	0.96
chr3:123415314	rs9814686	A	G	26.59	$4.7x10^{-8}$	1.16	0.59	0.91	$2.5x10^{-5}$	1.19	0.96
chr3:123417050	rs6783556	G	A	26.58	$4.7x10^{-8}$	1.16	0.59	0.91	$2.5x10^{-5}$	1.19	0.96
chr3:123416641	chr3:123416641:I	CTT	C	26.62	$4.9x10^{-8}$	1.16	0.60	0.91	$2.4x10^{-5}$	1.19	0.96
chr3:123413918	rs9884029	A	G	26.58	$4.9x10^{-8}$	1.16	0.59	0.91	$2.4x10^{-5}$	1.19	0.96
chr3:123415224	rs9814524	A	G	26.59	$5.1x10^{-8}$	1.16	0.59	0.91	$2.3x10^{-5}$	1.19	0.96
chr3:123411977	rs12489943	G	A	26.58	$5.1x10^{-8}$	1.16	0.59	0.91	$2.9x10^{-5}$	1.18	0.96
chr3:123415544	rs9856914	G	A	26.69	$5.4x10^{-8}$	1.16	0.72	0.94	$2.4x10^{-5}$	1.19	0.96

Supplementary Table 11 Conditional association analysis of the variants rs7627468 and rs73186030 at the *CASR* locus for kidney stones and serum calcium.

Position(Hg18)	SNP ID	Maf (%)	Covariate SNP ID	Unadjusted OR/Effect(SD%)	Unadjusted *P*	Adjusted OR/Effect(SD%)	Adjusted *P*	Phenotype
chr3:123428789	rs7627468†	26.80	rs73186030	1.16	$2.0{\times}10^{-8}$	1.16	$3.5{\times}10^{-8}$	Kidney stones
				1.6	$1.1{\times}10^{-3}$	2.2	$3.9{\times}10^{-6}$	Serum calcium
				2.5	$6.0{\times}10^{-3}$	3.1	$7.0{\times}10^{-4}$	Calcium inoized
chr3:123496155	rs73186030†	9.13	rs7627468	0.96	0.33	0.98	0.59	Kidney stones
				12.1	$2.0{\times}10^{-61}$	12.3	$9.6{\times}10^{-64}$	Serum calcium
				11.2	$1.1{\times}10^{-15}$	11.6	$1.5{\times}10^{-16}$	Calcium Ionized

†R^2= *0.0073*

Supplementary Table 12 The proportion of sibling recurrence risk explained by risk alleles of the kidney stone associated sequence variants identified.

					**Kidney Stones**				**Recurrent kidney stones**			
**SNP ID**	**Position(Hg18)**	**RA**	**RAF(%)**	**Gene**	***P***	**OR**	**$\Lambda_{S[i]}$**	**λ_S* *(%)***	***P***	**OR**	**$\Lambda_{S[i]}$]**	**λ_S** *(%)***
rs199565725	chr21:36757108	AAC	76.32	CLDN14	$4.7{\times}10^{-13}$	1.23	1.007	1.13	$3.5{\times}10^{-9}$	1.30	1.011	1.22
rs12654812	chr5:176726797	A	41.84	SLC34A1	$5.7{\times}10^{-11}$	1.18	1.007	1.13	$4.4{\times}10^{-7}$	1.21	1.009	1.00
rs1256328	chr1:21769354	T	17.79	ALPL	$5.8{\times}10^{-10}$	1.21	1.006	0.97	$4.0{\times}10^{-6}$	1.23	1.007	0.78
rs7627468	chr3:123428789	A	26.80	CASR	$2.0{\times}10^{-8}$	1.16	1.005	0.81	$4.1{\times}10^{-5}$	1.18	1.006	0.67
NA	chr5:176757439	G	0.46	SLC34A1	$8.5{\times}10^{-5}$	1.82	1.003	0.49	$2.8{\times}10^{-5}$	2.38	1.009	1.00
NA	chr7:142319969	C	0.13	TRPV5	$2.3{\times}10^{-3}$	2.17	1.002	0.32	$4.1{\times}10^{-5}$	3.62	1.009	1.00
Combined Λsib							1.030	4.81			1.052	5.66

$\lambda_{S[i]}$= sibling recurrence risk ratio, λ_{sib}(%) = is the proportion of sibling recurrence risk explained by risk variants calculated by the following equation $\log(\lambda_{S[i]})/\log(\lambda_S)$, RA = risk allele, RAF = risk allele frequency

* λ_S for kidney stones in Iceland was estimated at 1.85 by Edvardsson et al (PMID:19921989)

** λ_S for recurrent kidney stones in Iceland was estimated at 2.42 by Edvardsson et al (PMID:19921989)

Supplementary Table 13 The 50 most prevalent conditions among the 2,636 sequenced Icelanders.

**Disease**	**N**
Coronary Artery Disease	474
Chronic Kidney Disease	424
Obesity	394
Hypertension	371
Type 2 diabetes	307
Osteoporosis	260
Atrial Fibrillation	259
Myocardial Infarction	246
Alzheimer's disease	214
Asthma	211
Osteoarthritis	209
Urinary Tract Infection	208
Systemic Lupus Erythematous	207
Alcohol Dependence	199
Breast Cancer	196
Depression	171
Sleep Apnea	165
Autism Spectrum Disorders	160
Kidney Stones	160
Nicotine Dependence	158
Prostate Cancer	158
Gallstones	156
Schizophrenia	155
Attention Deficit Hyperactivity Disorder	154
Glaucoma	138
Mental Retardation	138
Colorectal Adenoma	134
Migraine	132
Age Related Macular Degeneration	127
Ischaemic Stroke	122
Psoriasis	120
Epilepsy	118
Tuberculosis	118
Basal Cell Carcinoma Of The Skin	115
Parkinson's Disease	108
Diverticular Disease	107
Cataract	106
Bronchitis	98
Tourette Syndrome	84
Heart Failure	81
Emphysema	80
Dyslexia	79
Hypothyroidism	79
Sick Sinus Syndrome	78
Peripheral Artery Disease	77

Rheumatoid Arthritis	73
Benign Prostatic Hyperplasia	70
Congenital Heart Disease	70
Hypertension In Pregnancy	69
Panic Disorder	69

Supplementary Table 14. The demographics of the sequenced, chip-typed and relatives of chip-typed individuals. [a]Year of birth. [b]Fraction currently alive. [c]Current age for the living. [d]Age at death.

Demographic	Sequenced	Chip-typed	Relatives of chip-typed
N	2,636	104,220	294,212
Female (%)	54.0	55.1	46.8
YOB[a] (SD)	1950 (23)	1953 (23)	1964 (36)
Alive[b] (%)	72.5	84.9	72.9
Age[c] (SD)	55 (20)	56 (70)	33 (20)
Lifespan[d] (SD)	79 (13)	80 (13)	59 (28)

Supplementary Table 15. Illumina SNP chips used in genotyping. Shown are the number of autosomal SNPs used in long range phasing and the number of PNs genotyped on each chip.

Chip	N autosomal LRP SNPs	N PNs
HumanHap300 (v1.0.0)	292,011	15,630
HumanHap300v2 A	292,918	6,656
HumanCNV370-Quadv3 C	299,544	288
HumanCNV370v1 C	303,859	13,673
Human610-Quadv1 B	408,023	13,380
Human1Mv1 C	542,300	544
HumanOmni2.5-8v1 A	554,797	4,114
HumanOmni2.5-4v1-Multi H	556,834	413
HumanOmni2.5-4v1 H	556,834	2,390
HumanOmniExpress-12v1-1 B	557,014	597
HumanOmniExpress-12v1-Multi H	559,377	2,818
HumanOmniExpress-12v1 H	559,377	31,674
HumanOmni1-Quad v1-0 B	560,775	10,882
Human1M-Duov3 B	565,537	471
HumanOmni5-4v1 B	631,692	690
